# Impacts of Carbon Dioxide Enrichment on Landrace and Released Ethiopian Barley (*Hordeum vulgare* L.) Cultivars

**DOI:** 10.3390/plants10122691

**Published:** 2021-12-07

**Authors:** Mekides Woldegiorgis Gardi, Waqas Ahmed Malik, Bettina I. G. Haussmann

**Affiliations:** 1 Institute of Cropping Systems and Modelling, University of Hohenheim, Fruwirthstrasse 14, 70599 Stuttgart, Germany; 2 Institute of Biostatistics, University of Hohenheim, 70599 Stuttgart, Germany; W.Malik@uni-hohenheim.de; 3 Institute of Plant Breeding, Seed Science and Population Genetics, University of Hohenheim, 70599 Stuttgart, Germany; bettina.haussmann@uni-hohenheim.de

**Keywords:** barley, biomass, CO_2_ enrichment, *Hordeum vulgare* L., water-use efficiency, yield

## Abstract

Barley (*Hordeum vulgare* L.) is an important food security crop due to its high-stress tolerance. This study explored the effects of CO_2_ enrichment (eCO_2_) on the growth, yield, and water-use efficiency of Ethiopian barley cultivars (15 landraces, 15 released). Cultivars were grown under two levels of CO_2_ concentration (400 and 550 ppm) in climate chambers, and each level was replicated three times. A significant positive effect of eCO_2_ enrichment was observed on plant height by 9.5 and 6.7%, vegetative biomass by 7.6 and 9.4%, and grain yield by 34.1 and 40.6% in landraces and released cultivars, respectively. The observed increment of grain yield mainly resulted from the significant positive effect of eCO_2_ on grain number per plant. The water-use efficiency of vegetative biomass and grain yield significantly increased by 7.9 and 33.3% in landraces, with 9.5 and 42.9% improvement in released cultivars, respectively. Pearson’s correlation analysis revealed positive relationships between grain yield and grain number (*r* = 0.95), harvest index (*r* = 0.86), and ear biomass (*r* = 0.85). The response of barley to eCO_2_ was cultivar dependent, i.e., the highest grain yield response to eCO_2_ was observed for *Lan_15* (122.3%) and *Rel_10* (140.2%). However, *Lan_13*, *Land_14*, and *Rel_3* showed reduced grain yield by 16, 25, and 42%, respectively, in response to eCO_2_ enrichment. While the released cultivars benefited more from higher levels of CO_2_ in relative terms, some landraces displayed better actual values. Under future climate conditions, i.e., future CO_2_ concentrations, grain yield production could benefit from the promotion of landrace and released cultivars with higher grain numbers and higher levels of water-use efficiency of the grain. The superior cultivars that were identified in the present study represent valuable genetic resources for future barley breeding.

## 1. Introduction

The global demand for food crops is increasing and may continue to do so for decades. A 70−100% increase in the cereal food supply by 2050 is required to feed the predicted world population of over nine billion people [1]. In terms of production and consumption, barley (*Hordeum vulgare* L.) is one of the most important cereal crops in the world following wheat, maize, and rice. It is cultivated both in highly productive agricultural systems and at the subsistence level in marginal environments [2]. Ethiopia is the second-largest barley producer in Africa, accounting for nearly 25% of the total production [3]. It has been cultivated in Ethiopia for the last 5000 years and accounts for 8% of the total cereal production in the country [4]. In the 2017/18 growing season, the national area coverage was 975,300 ha, with the production and productivity values of barley being approximately 2.1 million tons and 2.17 tons ha^−1^, respectively [3]. It is grown at elevations from 1500 to over 3500 m above sea level (m.a.s.l) and is predominantly cultivated between 2000 and 3000 m.a.s.l. [5,6].

Ethiopian barley germplasm has been used internationally as a source of useful genes due to its improved traits, including improved protein quality and disease and drought tolerance [5,7]. Long-term geographic isolation and adaptation to diverse climatic conditions and soil types resulted in a high level of variation between cultivars [8]. The crop is primarily used as a type of food and beverage in more than 20 different ways, which reflects its cultural and nutritional importance [9]. Despite its importance and morphological variations, one key challenge in barley breeding is the issue of developing cultivars that can face the challenges of changing climatic conditions [10]. Changes in the global atmospheric CO_2_ concentration constitute one of the most important and well-known examples of global climate change. The current increase in CO_2_ will likely continue into future decades and may bring the concentration close to 550 ppm by 2050 [11,12]. Elevated CO_2_ (eCO_2_) levels are known to have positive effects on photosynthetic processes, and consequently, on plant growth in C3 plant species, mainly through the modification of water and nutrient turnover [13,14,15]. Thus, as CO_2_ is fundamental for plant production, understanding cultivar behavior and the targeted exploitation of this resource via plant breeding could optimize yields and contribute to future food security [16,17,18].

Several CO_2_-enrichment studies regarding major cereal species, i.e., barley [19,20,21], wheat [22,23], and rice [24], reported substantial intraspecific variation between cultivars regarding plant growth and yield in response to eCO_2_ enrichment. In contrast, another study regarding different cultivars of wheat reported non-significant intraspecific variation in yield responses [25]. To the best of our knowledge, no information is currently available regarding the response of Ethiopian barley cultivars to eCO_2_. Therefore, the present study aimed to evaluate the growth, yield formation, and water-use efficiency response of Ethiopian barley cultivars under current and future CO_2_ concentrations.

## 2. Results

### 2.1. Plant Height and Biomass Allocation Pattern

**Figure 1:**
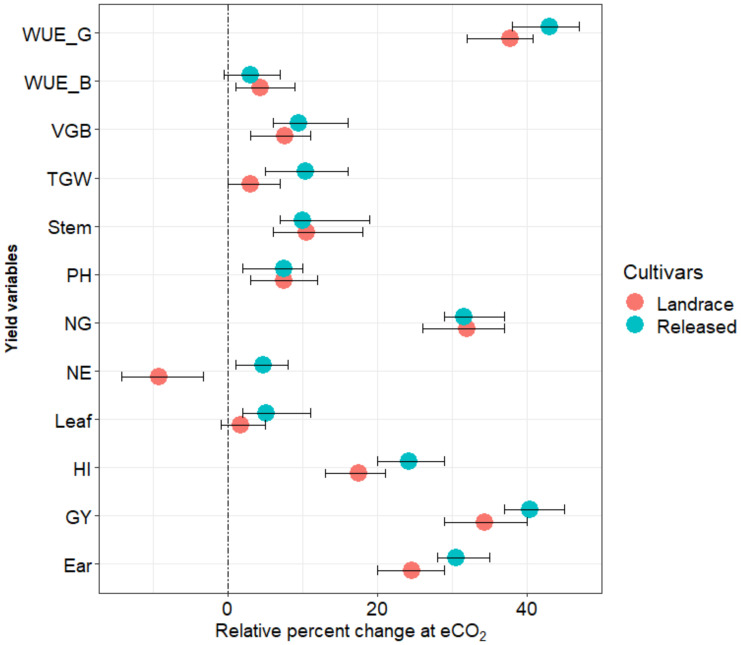
Relative effect of eCO_2_ condition on plant height, biomass fractions, yield components, and water-use efficiency of barley. Average relative changes due to CO_2_ enrichment against aCO_2_ are presented, with error bars representing their standard errors. Ear: ear biomass; GY: grain weight; HI: harvest index; Leaf: leaf biomass; NE: number of ears; NG: grain number; PH: plant height; Stem: stem biomass; TGW: thousand-grain weight; VGB: vegetative biomass; WUE_B: water-use efficiency of vegetative biomass; and WUE_G: water-use efficiency of grain.

**Figure 2:**
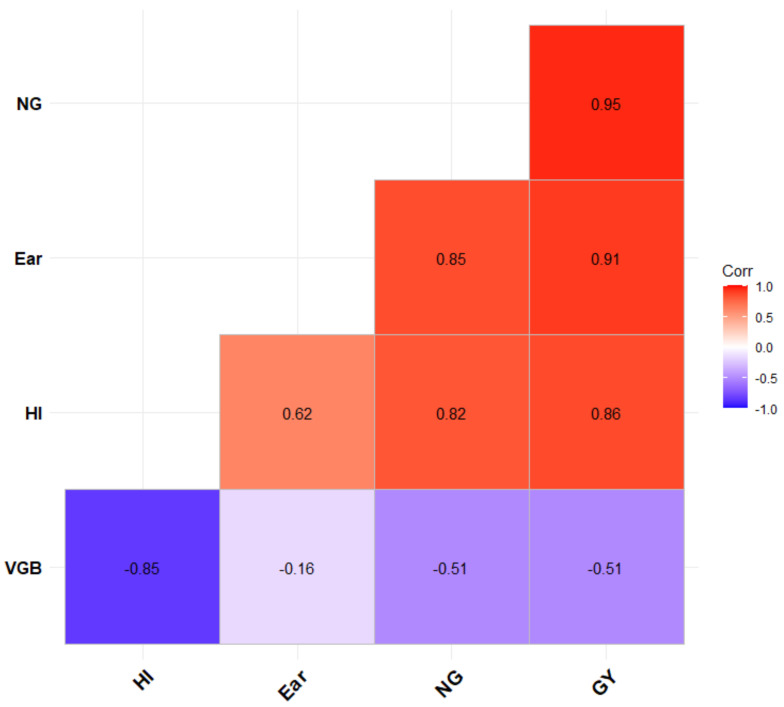
Correlation between grain yield, yield parameters, and water-use efficiency. VGB: vegetative biomass; Ear: ear biomass; NG: number of grains; GY: grain yield; WUE_G: water-use efficiency of grains; HI: harvest index. The value shows Pearson’s correlation coefficient. The minus sign indicates a negative correlation between the variables.

Significant impacts caused by CO_2_ enrichment were observed for several yield variables in both the landrace and released cultivars, except in the variables of leaf biomass fraction, the number of ears per plant, and thousand-grain weight. The interaction between CO_2_ and the cultivars also had a significant effect on most of the yield variables (Table 1). The average plant height of the landrace and released cultivars in the ambient CO_2_ (aCO_2_) condition were 101.9 and 94.5 cm, respectively (Table 1). The effect of CO_2_ enrichment was observed in the variable of plant height, with an increase of 7.6% in landraces and 6.7% in released cultivars (Figure 1). The average vegetative biomass of the landrace was 35.6 g dry weight per plant in the aCO_2_ condition (Table 1), while the released cultivars had 39.4 g dry weight per plant (Table 1). Significant increases in vegetative biomass, by 7.6 and 9.4%, respectively, were recorded across the landrace and released cultivars in the eCO_2_ condition (Figure 1). The increase observed in vegetative biomass was mainly due to the significant effect of eCO_2_ on the stem biomass in both the landrace and released cultivars (Table 1). As shown in Figure 2, a negative correlation between vegetative biomass and grain yield (*r* = −0.51, *p* < 0.05) as well as harvest index (*r* = −0.85, *p* < 0.001) was observed.

### 2.2. Grain Yield Parameters

Grain yield and its parameters were significantly affected by genotype/cultivars, CO_2_ treatment, and their interaction in both the landrace and released cultivars (Table 1). The average grain yield of the landrace was 8.1 g dry weight per plant, resulting from 13.8 ears and 146 grains per plant. On the other hand, the released cultivars had a grain yield of 6.7 g dry weight per plant from 12.8 ears and 134 grains per plant, on average, under the aCO_2_ conditions. Increases in the grain yield of the landrace and released cultivars, by 34.1 and 40.6%, respectively, were recorded under the eCO_2_ condition (Table 1 and Figure 1). All yield components contributed significantly to the increase in grain yield, except for the number of ears. The number of grains per plant showed the largest increase of 32.2% in the landrace and 31.3% in the released cultivars (Table 1 and Figure 1). In accordance with this, the harvest index increased by 14.3% (landraces) and 23.3% (released cultivars) in the eCO_2_ condition. The eCO_2_ condition was recorded to have a significant effect on thousand-grain weight for the released cultivars; the thousand-grain weight increased by 10.4% on average, while the change was not significant in the landrace (Figure 1).

**Figure 3:**
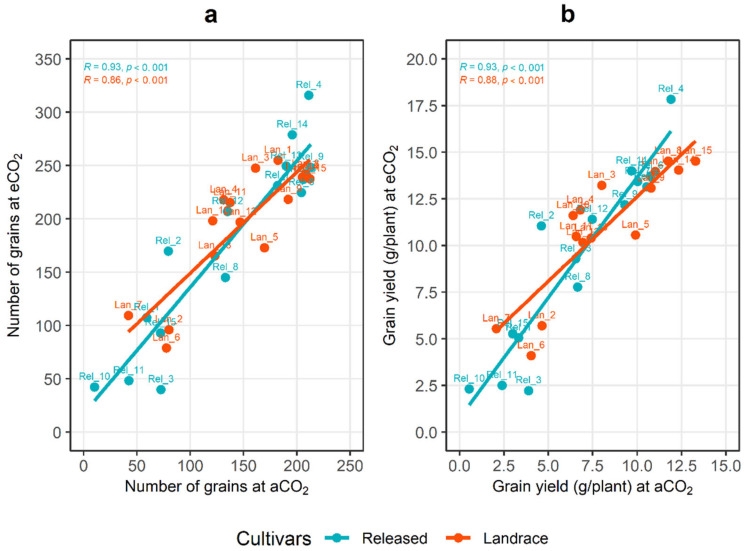
Mean response of landrace and released cultivars under elevated (500 ppm) CO_2_ plotted against mean response under ambient (400 ppm) CO_2_, where responses refer to (**a**) number of grains per plant and (**b**) grain yield (in grams) per plant.

In Figure 2, the correlation analysis revealed that grain yield had a positive and strong association with the number of grains (*r* = 0.95, *p* < 0.001), ear biomass (*r* = 0.91, *p* < 0.001) and harvest index (*r* = 0.86, *p* < 0.001). In addition, the performance of the genotypes/cultivars regarding the response of grain yield under the aCO_2_ condition versus the eCO_2_ condition had a significant and positive correlation in the landrace (*r* = 0.64, *p* = 0.01) and released cultivars (*r* = 0.93, *p* < 0.001), as shown in Figure 3. Among the landrace cultivars, *Lan_15* displayed the highest yield, while *Lan_7* displayed the lowest yield under both the ambient and elevated CO_2_ conditions. Comparing the released cultivars, the highest grain yield was recorded for *Rel_4*, and *Rel_10* had the lowest yield. Moreover, a strong and positive correlation of cultivars was recorded for grain number per plant under the aCO_2_ condition versus the eCO_2_ condition (Figure 3); however, the best genotypes under aCO_2_ were not always the best genotypes under eCO_2_ in terms of both number of grains and grain yield.

### 2.3. Water-Use Efficiency

**Figure 4:**
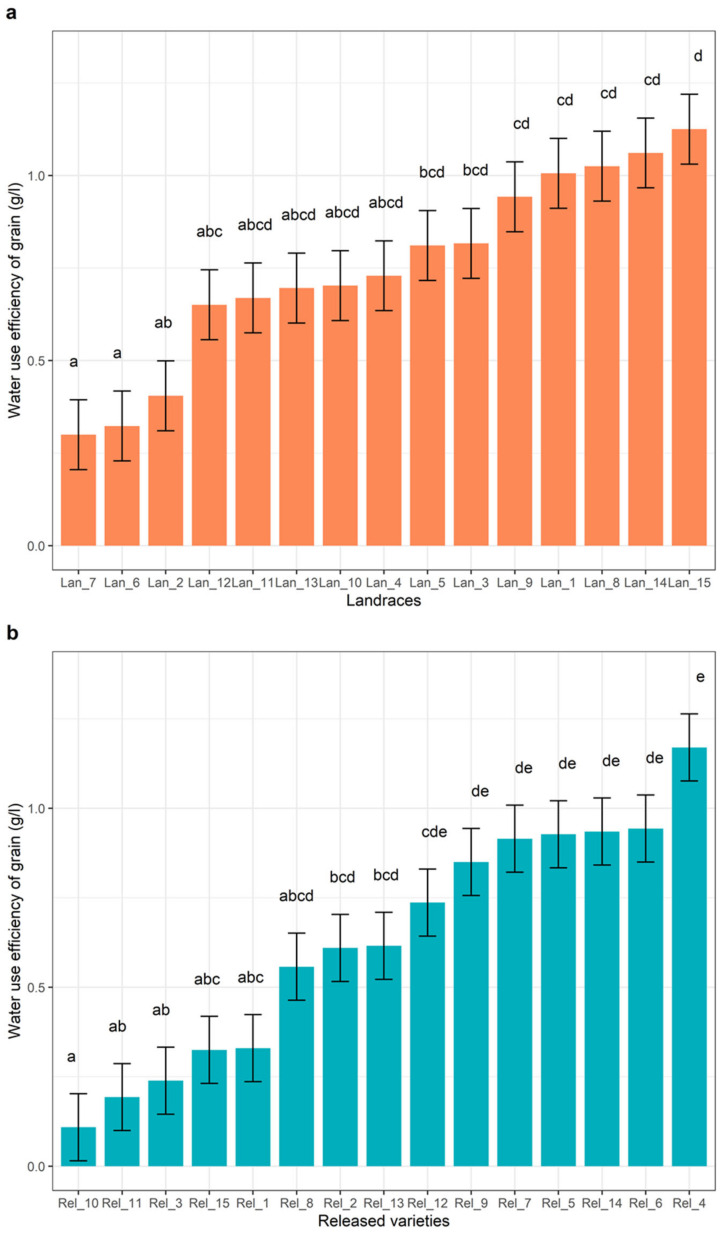
Mean response of landrace (**a**) and released cultivars (**b**) regarding the water-use efficiency of grains (WUE_G, g L^−1^). The letters indicate the significant level between genotypes/cultivars. Mean values sharing a letter are not significantly different.

The variables of water-use efficiency of vegetative biomass (WUE_B) and grain (WUE_G) were significantly affected by the CO_2_ condition and type of cultivar (*p* < 0.001), as shown in Table 1. However, their interaction did not affect the response of total water use in both the landrace and released cultivars. In the aCO_2_ condition, the landrace cultivar used 9.2 L plant^−1^ of WU_T, and had 4.7 g L^−1^, WUE_B, and 0.9 g L^−1^ WUE_G (Table 1). On the other hand, the released cultivars used 9.3 L plant^−1^ of WU_T and had 4.9 g L^−1^ WUE_B, and 0.7 g L^−1^ WUE_G (Table 1). The levels of total water consumption of water by the landrace and released cultivars were not significantly different under the different CO_2_ levels. The effect of CO_2_ enrichment was higher in the response of WUE_G than WUE_B. WUE_G was increased by 33.3% in landraces and 42.9% in the released cultivars (Table 1 and Figure 1). In comparison, *Lan_15* and *Rel_4* showed the highest WUE_G among the landrace and released cultivars, respectively, while the lowest WUE_G was observed in *Lan_6*, *Lan_7,* and *Rel_10* (Figure 4a,b).

## 3. Discussion

### 3.1. The Overall Effect of eCO_2_ on Vegetative Biomass, Grain Yield, and Water-Use Efficency

Atmospheric CO_2_ enrichment is expected to contribute to the required increase in grain yield production in the future [15,26,27]. Our findings from the climate chamber experiment, where the eCO_2_ condition was applied as a single factor, correspond well with findings in previously published data. In the present study, on average, vegetative biomass was increased by 7.6% in landraces and 9.4% in the released cultivars, respectively. The enhancement was predominantly due to higher biomass allocation towards ear and stem biomass. The eCO_2_ condition was observed to have a significant effect on the response of leaf biomass in the released cultivars alone. In line with the present results, findings from CO_2_ enrichment studies regarding barley reported the significant enhancement of vegetative biomass due to higher CO_2_ concentrations [28,29,30]. A previous study [15] summarized the biomass response of the C3 species and reported an average enhancement of vegetative biomass by 16% under eCO_2_ conditions. Comparable results were also reported regarding other C3 crops, such as wheat [31] and rice [26].

In the present study, the released cultivars had a higher relative grain yield increase (40.6%) under the eCO_2_ condition as compared to the landraces (34.1%). This supports the hypothesis that enhanced net-photosynthesis in eCO_2_ conditions was unconsciously targeted through breeding. However, surprisingly, the landrace group had higher actual grain yield production levels under both the aCO_2_ and eCO_2_ conditions. In support of this finding [28], grain yield was determined via grain number per plant and ear biomass, which indicates that CO_2_ enrichment and the acquisition of extra carbon were carried forward to the grains rather than the biomass yield. Previous studies regarding barley [19,20] and wheat [32,33] reported the positive correlation of grain yield with grain number. In the current study, an average enhancement of thousand-grain weight by 10.4% due to eCO_2_ conditions was recorded in the released cultivars, whereas the response was not significantly affected in the landraces. In line with our findings, a study regarding wheat reported an enhancement of thousand-grain weight by 3.8–7.0% [34]; on the other hand, a non-significant effect of eCO_2_ conditions on the thousand-grain weight of barley and wheat was reported in other studies [20,35]. The effects of eCO_2_ conditions on the harvest index have been reviewed in rice, wheat, and soybean, with contradictory results. In the present study, the harvest index was increased by 23.3 and 14.3% in the released and landrace cultivars, respectively, under the eCO_2_ condition. Similarly, in [27], a significant increase in the harvest index was also displayed in rice under eCO_2_ conditions, which was contrary to a decrease in harvest indexes related to soybean and wheat [26,36]. The actual grain yield of landrace observed in the present study was higher compared to that of the released cultivars; however, the positive effect of eCO_2_ was greater in the released cultivars. Accordingly, the relative percentage change of the harvest index was observed to be higher for the released cultivars compared to the landraces. Our finding supports the effort of breeding to reduce the percentage of vegetative biomass to increase the harvest index of crops, which is in line with the findings of [17].

As CO_2_ levels rise above the current ambient level, photosynthesis is commonly enhanced and transpiration is frequently reduced, resulting in greater water efficiency and increased plant growth and productivity [37]. In the present study, a significant improvement regarding WUE_G was displayed. Average enhancements in the values of WUE_G by 33.3 and 42.9% were observed in the landrace and released cultivars, respectively, under the eCO_2_ condition. In agreement with these findings, previous studies reported that eCO_2_ conditions had a significant effect on the WUE_G and WUE_B values of barley and other crops. For instance, a study regarding two barley cultivars reported a significant enhancement of water-use efficiency of vegetative biomass and grain under well-watered conditions [17]. Furthermore, increases in WUE values by 20% under well-watered and by 42% under drought conditions, due to the presence of eCO_2_, were reported [29]. Regarding wheat, the authors of [38,39] reported a significant enhancement of WUE_B and WUE_G values due to high eCO_2_ conditions. On the other hand, the author of [40] revealed a clear reduction in the water consumption of barley under eCO_2_ conditions. The current study, as well as several previous studies, revealed that eCO_2_ conditions cause increases in water-use efficiency values by increasing growth and yield more so than by increasing water consumption. This would be beneficial for use in future food production, especially in water-limited areas.

### 3.2. Cultivar Specific Responses to eCO_2_ on Barley Production

In this study, a wide range of intraspecific variation was observed in the responses of the measured yield parameters to the eCO_2_ condition, from negative to large increments. The response of grain yield to the eCO_2_ condition ranged from −25% (*Lan_14*) to +122.3% (*Lan_15)* in the landraces, while the released cultivars showed a 42% reduction in grain yield (*Rel_3*) to an increment of 140.2% (*Rel_10*) under the eCO_2_ condition. High grain yield and stability were found among landraces and the released cultivars. The landraces originated and were grown in different altitudes, indicating that suitable resources for climate resilience are available from different areas. The highest yielding landraces were *Lan_15*, *Lan_8*, *Lan_1*, *Lan_9,* and *Lan_6* under both the aCO_2_ and eCO_2_ conditions. The highest yielding landraces were grown in various parts of Ethiopia between 1642 and 3570 m.a.s.l, indicating the diversity and potential of choosing cultivars for future climate conditions. On the other hand, the highest yielding released cultivars were *Rel_4*, *Rel_5*, *Rel_6*, *Rel_7,* and *Rel_10*, which were characterized by early maturation, high yields, and resistance to lodging and leaf diseases (*Pyrenophora teres* and *Rhynchosporium secalis*). As shown in our findings, CO_2_ enrichment studies regarding different barley cultivars reported a significant variation among cultivars in the response of grain yield and its parameters [20,21,41]. The greater enhancement of ear biomass per plant and improvement regarding WUE_G values significantly contributed to the observed grain yield gain in the highest yielding cultivars. In line with these findings, several studies have reported that barley yield responses to eCO_2_ conditions are mostly cultivar dependent [19,23,42]. Studies involving other C3 crops have also reported significant differences between cultivars tested in future climate change scenarios. Variations in the responses to eCO_2_ conditions in rice cultivars, for example, have been recorded, ranging from a 31% yield reduction to a 41% yield gain [24,43]. Similarly, significant variation in yield response under eCO_2_ conditions, ranging from 20 to 80%, was observed in soybean cultivars [44]. Further variations in yield response were observed in other studies, with yield gains of between 31 and 41% being found [24,43]. As has been seen in previous studies, in the present study, negative growth effects of eCO_2_ were observed regarding vegetative biomass and grain yield. The negative yield responses may partly be associated with alterations in the shoot: root carbon allocation between the cultivars examined. Previous studies reported positive root growth effects in barley via eCO_2_ conditions [45,46]. Cultivars with negative vegetative biomass accumulation under eCO_2_ were allotted newly assimilated carbon, but this would preferentially take place below the ground level for the enhanced development of their root systems at the expense of the vegetative biomass [21]. A review of different experiments conducted under eCO_2_ conditions listed 13 C3-plant species that exhibited reductions in vegetative biomass by up to 42% [47]. A set of more than 100 spring barley cultivars grown under eCO_2_ conditions yielded negative responses comparable to the current findings [48]. In general, studies on C3 crops indicate that intraspecific yield variations under eCO_2_ conditions are primarily related to changes in carbon allocation within cultivars, rather than physiological traits related to carbon assimilation [45,46]. The current study, as well as other similar studies, have found a wide range of eCO_2_ responsiveness in some of the world’s most important food crops, implying that selecting for eCO_2_ responsiveness may ensure long-term productivity under eCO_2_ conditions [18,26,49,50]. The *Lan_15*, *Lan_8*, *Lan_1*, *Lan_9*, and *Lan_6* variants among the landraces and the *Rel_4*, *Rel_5*, *Rel_6*, *Rel_7*, and *Rel_10* variants among the released cultivars are the top five highest-yielding variants due to improved grain number values under the eCO_2_ condition. They represent important genetic resources for use in future barley breeding programs. Despite the overall positive correlation of genotypes/cultivars, the best genotypes under aCO_2_ might not always be the best genotypes under eCO_2_; thus, direct selection under eCO_2_ is needed to identify the best varieties for future climates.

## 4. Materials and Methods

### 4.1. Genetic Material and CO_2_ Enrichment

**Figure A1:**
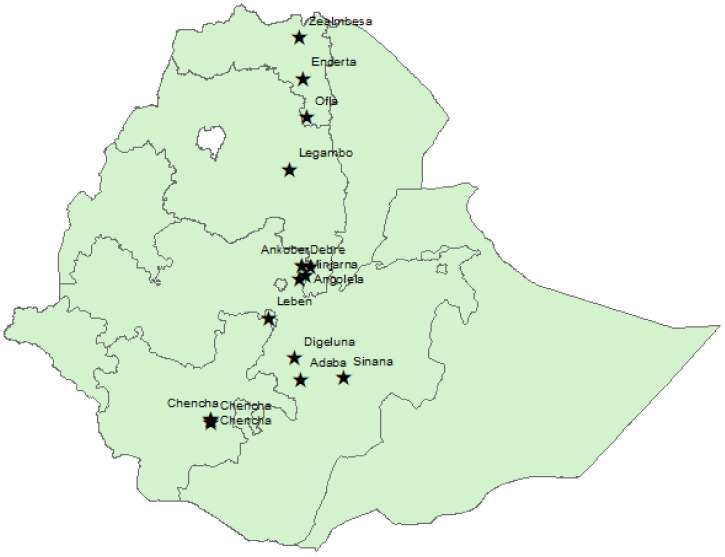
Map of origin of Ethiopian landrace cultivar collection.

Thirty Ethiopian barley cultivars consisting of 15 landraces and 15 released cultivars were obtained from Holetta Agricultural Research Centre (HARC) in Ethiopia. The landraces represent dominant barley landraces that are cultivated in different parts of Ethiopia. The released cultivars were chosen based on their diversity regarding adaptation and genetic background. They were released from 1975, are grown in different parts of the country, and differ in their traits such as grain yield (Figure A1, Table A1 and Table A2). The cultivars were cultivated in six identical climate chambers (Vötsch BioLine, Balingen, Germany) in which the climatic variables could be controlled. To mimic a realistic seasonal climate within the climate chambers, the daily temperature and relative humidity mean of Holeta from the period 2008–2018, and which are registered at World Weather Online (https://www.worldweatheronline.com, accessed on 12 January 2019), was used. In total, 27 weekly climate profiles were derived from these 10-year time series, representing the main growing season in Ethiopia. The day length (12 h) and the daily temperatures (daily mean of the coldest week: 8 °C; daily mean of the warmest week: 25 °C) were adapted. The CO_2_ concentration within the chambers did not follow any time course but was set to constant values of 400 ppm in three chambers (ambient concentration, aCO_2_) and 550 ppm in another three chambers (elevated concentration, eCO_2_).

### 4.2. Plant Cultivation and Measurement of Plant-Related Parameters

The polyvinyl chloride pots used in the experiment were 40 cm in height and 10.3 cm in diameter, with a total volume of 3.33 L and a surface area of 83.33 cm^2^. These pots were filled with 3.3 kg of sand and standard soil (Fruhstorfer Erde LD80, Hawita GmbH, Vechta, Germany) with a 2:1 ratio. The standard soil, LD80, comprised 50% peat, 35% volcanic clay, and 15% bark humus, and it was enriched with slow-releasing fertilizers. The pH (CaCl_2_) of the medium was 5.9, the organic matter content was 35% (loss-on-ignition), and the salt content was 1 g L^−1^ KCl. The nutrient availability of the LD80 standard medium was (mg L^−1^) 150 N, 150 P_2_O_5_, and 250 K_2_O. Per cultivar, five seeds were grown and thinned at the seedling stage in two experimental plants per pot. Once a week, pots and CO_2_ treatments were rotated between chambers to avoid any potential chamber effects. Plants were watered with 500 mL at the beginning of the experiment and were regularly watered throughout with an adequate amount to avoid drought. Pots were weighed once a week and adjusted to a weight of 5 kg to monitor differences in the water consumption of plants from different CO_2_ treatments over time. The total water consumption ranged between 8.6 and 9.7 L in landraces and between 8.7 and 9.8 L in released cultivars. The values of total water use (WU_T, Equation (1)), water-use efficiency of vegetative biomass (WUE_B, Equation (2)), and water-use efficiency of grain yield (WUE_G, Equation (3)) were calculated.

When the plants reached full maturity, plant height and total pot weight were measured before harvesting. Afterward, plants were harvested and separated into the vegetative biomass fractions (leaves, stems, and reproductive organs/ears). The single plant fractions were oven-dried at 30 °C (reproductive organs/ears) and 60 °C (stems and leaves) until they reached a constant weight before their dry weight was determined. The share to which single plant fractions contributed to total plant biomass was calculated and given as leaf, stem, and ear dry matter weight per plant. Grains were removed from the ears by manual threshing to determine the total grain yield, thousand-grain weight, and grain number, as well as the harvest index per plant.

(1) $$WU_{T}=\frac{Total\;water\;applied\;\left(L\right)}{Plant}$$

(2) $$WUE_{B}=\frac{Biomass\;yield\;\left(g\right)}{Total\;water\;applied\;\left(L\right)}$$

(3) $$WUE_{G}=\frac{Grain\;yield\;\left(g\right)}{Total\;water\;applied\;\left(L\right)}$$

### 4.3. Statistical Analyses

The experiment was conducted using a randomized split-plot design with three replicates per CO_2_ treatment level; the CO_2_ treatment level was used as the main plot factor. The two levels of CO_2_ were randomly assigned to a climate chamber, and cultivars were randomly placed in a climate chamber. Once a week, pots and CO_2_ treatments were rotated between chambers to avoid any potential chamber effects. Following the experimental design, a two-way analysis of variance (ANOVA) was applied to test the significance of the main effects of genotype/cultivar and CO_2_ treatments, as well as their interactions regarding both the landrace and released cultivars. In addition, the main effects of altitude and its interaction with CO_2_ levels were analyzed regarding the landrace. Means were separated using Tukey HSD post hoc tests. Pearson’s correlation coefficients were calculated to compare response variables and the performance of cultivars under the aCO_2_ condition versus the eCO_2_ condition. All the analyses were performed using the R programming language, version 4.0.1 [51].

## 5. Conclusions

Elevated CO_2_ is beneficial to barley growth, yield, and water-use efficiency. The present study evaluated thirty Ethiopian barley cultivars and showed that eCO_2_ levels provoke a significant enhancement of vegetative biomass and grain yield values. In comparison, grain yield was much more responsive to the eCO_2_ condition than vegetative biomass, mainly due to a significant enhancement of the ear biomass value, grain number, and harvest index. The water-use efficiency of vegetative biomass and the water-use efficiency of grain was enhanced in future climate condition. The grain yield gain was positively associated with the high grain number and water-use efficiency of grain per plant. On average, the released cultivars benefited more from CO_2_ fertilization than the landraces. However, a wide range of intraspecific variation was observed within the responses of biomass and grain yield parameters across both the landrace and released cultivars. For instance, the cultivars *Lan_15* and *Rel_4* were the highest yielding variants among the landrace and released cultivars, respectively, under the current and future CO_2_ levels and represent important genetic resources for use in the future barley breeding in Ethiopia. The investigation of the interaction between cultivar types and the environment could help to better understand the thresholds for cultivars’ performance under climate change conditions. Grain yield production under future climate conditions could benefit from the identification of cultivars with higher grain numbers and more efficient water use in grain. However, food security involves more than just production. Further attention is required regarding the investigation of the nutritional quality of barley cultivars under eCO_2_ conditions. Moreover, the growth and stress tolerance values of Ethiopian barley cultivars in response to the interactive effects of eCO_2_ conditions, warming, and drought should be examined in order to achieve better exploitation of germplasm resources under changing climatic conditions.

## Figures and Tables

**Table 1:** Analysis of variance results. Mean and standard error (S.E.) of phenological parameters of landrace (Gen) and released cultivars (Cul) under ambient and elevated CO_2_ conditions, as well as their interactions.

Variables	Cultivar	aCO_2_	eCO_2_	S.E.	Δ %	CO_2_	Gen/Cul	CO_2_×Gen/Cul
Plant height (cm)	Landrace	101.9	109.6	3.8	7.6	***	*	*
	Released	94.5	100.8	3.8	6.7	***	***	ns
Vegetative biomass (g plant^−1^)	Landrace	35.6	38.3	2.0	7.6	***	***	***
	Released	39.4	43.1	2.0	9.4	***	***	***
Stem biomass (g plant^−1^)	Landrace	19.3	21.4	1.4	10.9	***	***	***
	Released	21.5	23.7	1.3	10.2	***	***	***
Leaf biomass (g plant^−1^)	Landrace	11.2	11.4	0.7	1.8	ns	***	***
	Released	12.6	13.3	0.7	5.6	***	**	*
Ear biomass (g plant^−1^)	Landrace	13.2	16.5	1.8	25.0	***	***	ns
	Released	11.9	15.6	1.8	31.1	***	**	ns
Chaff (awn) biomass (g plant^−1^)	Landrace	5.1	5.5	1.6	9.0	**	***	***
	Released	5.2	6.1	1.2	17.6	***	***	***
Number of ears (plant^−1^)	Landrace	13.8	15.2	1.8	10.2	ns	**	ns
	Released	12.8	13.4	2.2	4.7	ns	*	ns
Number of grain (plant^−1^)	Landrace	146.0	193.0	30.9	32.2	***	***	***
	Released	134.0	176.0	34.2	31.3	***	***	ns
Grain yield (g plant^−1^)	Landrace	8.1	10.9	1.7	34.1	***	***	***
	Released	6.7	9.42	1.7	40.6	***	***	**
Thousand-grain weight (g)	Landrace	54.5	56.2	3.1	3.1	ns	***	ns
	Released	49.2	54.3	5.6	10.4	*	**	ns
Harvest index	Landrace	0.21	0.24	0.03	14.3	**	***	ns
	Released	0.16	0.20	0.03	23.3	***	***	***
Total water use (WU_T, L plant^−1^)	Landrace	9.2	9.1	0.1	−1.1	*	***	***
	Released	9.3	9.3	0.1	0.0	ns	***	***
Water-use efficiency of vegetative biomass (WUE_B, g L^−^^1^)	Landrace	3.8	4.1	0.1	7.9	***	***	***
	Released	4.2	4.6	0.1	9.5	***	***	***
Water-use efficiency of grains (WUE_G, g L^−^^1^)	Landrace	0.9	1.2	0.1	33.3	***	***	ns
	Released	0.7	1.0	0.1	42.9	***	***	ns

Significance level: *p* < 0.001 (***); *p* < 0.01 (**); *p* < 0.05 (*); and non-significant (ns).

## Data Availability

The data presented in this study are available upon request from the corresponding author.

## Appendix A

**Table A1:** List of Ethiopian landrace cultivars and the origin of the collection.

Code	Cultivars	Region	Zone	Woreda	Latitude	Longitude	Altitude
Lan_1	215217-A	Amara	Debub	Legambo	11-39-00-N	39-00-00-E	3570
Lan_2	18330-A	Amara	Semen	Angolela	09-18-00-N	39-32-25-E	3325
Lan_3	219578-A	Amara	Semen	Debre	09-37-00-N	39-25-00-E	2690
Lan_4	243410	Amara	Semen	Ankober	09-36-00-N	39-44-00-E	2350
Lan_5	237021	Amara	Semen	Minjarna	09-10-20-N	39-20-00-E	1750
Lan_6	208816-A	Oromiya	Bale	Adaba	07-00-20-N	39-23-30-E	3500
Lan_7	237015	Oromiya	Arssi	Digeluna	07-45-00-N	39-11-00-E	2600
Lan_8	64233-C	Oromiya	Bale	Sinana	07-04-00-N	40-14-00-E	2460
Lan_9	18327	Oromiya	Semen	Leben	08-28-00-N	38-56-59-E	1642
Lan_10	216997	SNNP	Semen	Chencha	06-17-00-N	37-35-00-E	3030
Lan_11	208845	SNNP	Semen	Chencha	06-15-00-N	37-35-00-E	2850
Lan_12	234307	Tigray	Misrak Awi	Zealmbesa	14-16-00-N	39-21-00-E	3100
Lan_13	234293	Tigray	Debub Awi	Ofla	12-48-00-N	39-35-00-E	2410
Lan_14	237339	Tigray	Debub Awi	Enderta	13-30-00-N	39-28-00-E	2240
Lan_15	221325	SNNP	Semen	Chencha	06-09-00-N	37-36-00-E	2150

**Table A2:** List of Ethiopian released cultivars and their desired trait.

Code	Cultivars	Genetic Background/Pedigree	Year of Released	Desirable Traits of the Cultivars Other than Yield
Rel_1	Gobe	IICARDA germplasm-CBSS96Moo487T-D-1M-1Y-2M-oY	2012	
Rel_2	Cross 41/98	(50-16/3316-03)//(HB42/Alexis)	2012	High yielding, late maturing
Rel_3	EH 1493	White Sasa/Comp29//White Sasa/EH538/F2-12B-2	2012	High yielding, late maturing
Rel_4	EH1847	EH1847/F4.2P.5.2 (Beka/IBON64/91)	2011	
Rel_5	Bekji-1	EH 1293/F2-18B-11-1-14-18	2010	
Rel_6	HB-1307	EH-1700/F7. B1.63.70	2006	High yield, lodging resistant, resistant to leaf diseases (Pyrenophora teres and Rhynchosporium secalis), good biomass yield, and white seeded
Rel_7	Misccal-21	Azafran = Shyri//Gloria/Copal/3/Shyri/Grit; CMB87.643-2A	2006	High yield with good malting quality; resistance to lodging with multiple disease resistance
Rel_8	Meserach	Pure line selection- Kulumsa1/88	1998	Early maturing and tolerant to major leaf diseases (Pyrenophora teres and Rhynchosporium secalis)
Rel_9	HB-42	EIAR cross-IAR-H-81/comp29//comp14-20/coast	1984	Resistant to scalding (Rhynchosporium secalis) and good biomass yield
Rel_10	IAR/H/485	Pure line selection from local landrace in Arsi	1975	
Rel_11	Ardu 12-60B	Pure line selection from local landrace in Arsi	1986	
Rel_12	Balemi	Dominant farmers varieties in West shoa	1970	Tolerant to low soil fertility and drought, good flour quality
Rel_13	HB-1964	RECLA78//SHYRI/GRIT/3/ATAH92/GOB	2016	
Rel_14	HB-1965	Awra gebs X IBON64/91	2017	
Rel_15	HB-1966	CARDO/CHEVRON-BAR CBSS 96 WM 00019s	2017	
